# Complete exhaustion of dissolved nutrients in a large lowland river

**DOI:** 10.1007/s10661-024-12834-5

**Published:** 2024-06-25

**Authors:** Norbert Kamjunke, Tina Sanders

**Affiliations:** 1 Department of River Ecology, Helmholtz Centre for Environmental Research UFZ, Brückstraße 3a, 39114 Magdeburg, Germany; 2 Department of Aquatic Nutrient Cycles, Institute of Carbon Cycles, Helmholtz Zentrum Hereon, Max-Planck-Str. 1, 21502 Geesthacht, Germany

**Keywords:** Phytoplankton, Phosphorus, Nitrate, Silicate, Denitrification, Nitrate stable isotopes

## Abstract

Riverine phytoplankton takes up phosphate, dissolved silicate, and nitrate. We investigated which nutrients are depleted during a Lagrangian sampling in the free-flowing part of the River Elbe in 2023. As part of this study, we tested the hypotheses that nutrient depletion might be caused by (1) above-average phytoplankton biomass or by (2) decreased nutrient load of the river during previous years. Phytoplankton biomass increased up to 350 km in rivers and stopped increasing exactly when soluble reactive phosphorus had been completely consumed, and molar carbon to phosphorus ratios of seston indicated the beginning phosphorus limitation. The concentrations of dissolved silicate and nitrate dropped below the detection limit as well. In contrast to the results from eight previous longitudinal samplings taken in 2018–2022, nitrate exhaustion was detected for the first time in 2023 within the transect. This was caused neither by an above-average phytoplankton biomass nor by a declined overall nutrient load of the river in 2018–2023. Instead, denitrification appears to be the most plausible explanation for the downstream decrease of nitrate and the loss of total nitrogen which was supported by enrichment of nitrate stable isotopes and a decreasing ratio of nitrate ^15^N/^18^O.

## Introduction

The development of phytoplankton is a common phenomenon in large rivers and was described already in the River Continuum Concept (Vannote et al., 1980). Starting from the source, cells require a few days to multiply until they reach maximum densities in the middle section of the rivers (Reynolds & Descy, 1996). In the lowland, downstream, and estuarine parts of rivers, on the other hand, water depth and turbidity often limit light penetration and hence primary production. In order to multiply, phytoplankton needs dissolved nutrients such as phosphate, nitrate, and also silicate for diatom frustules. Thus, the concentrations of these nutrients often show a longitudinal decrease inversely to the increase in phytoplankton biomass. A depletion of phosphate and silicate has already been observed in several rivers such as the River Elbe (Rode et al., 2007), River Thames (Bowes et al., 2012), and River Loire (Minaudo et al., 2015). However, nitrate depletion has been reported at the transition to the estuary so far (Schulz et al., 2023) but not in the freshwater river itself.

Here, we investigated longitudinal phytoplankton and nutrient dynamics in the free-flowing part of the River Elbe (Germany) using a Lagrangian approach (i.e., according to travel time). In particular, we tested which nutrients become depleted in the river where primary production is dominated by phytoplankton (Deutsch et al., 2009) and densities of benthic grazers are negligible (Hardenbicker et al., 2016). In addition, we determined nitrate stable isotopes as indicators for autotrophic assimilation or heterotrophic denitrification (Schulz et al., 2023). To investigate potential reasons for nutrient decline, we tested the hypotheses that nutrient depletion might be caused by either (1) very high phytoplankton biomass compared to previous years or by (2) decreased nutrient load of the river during previous years.

## Material and methods

**Fig. 1:**
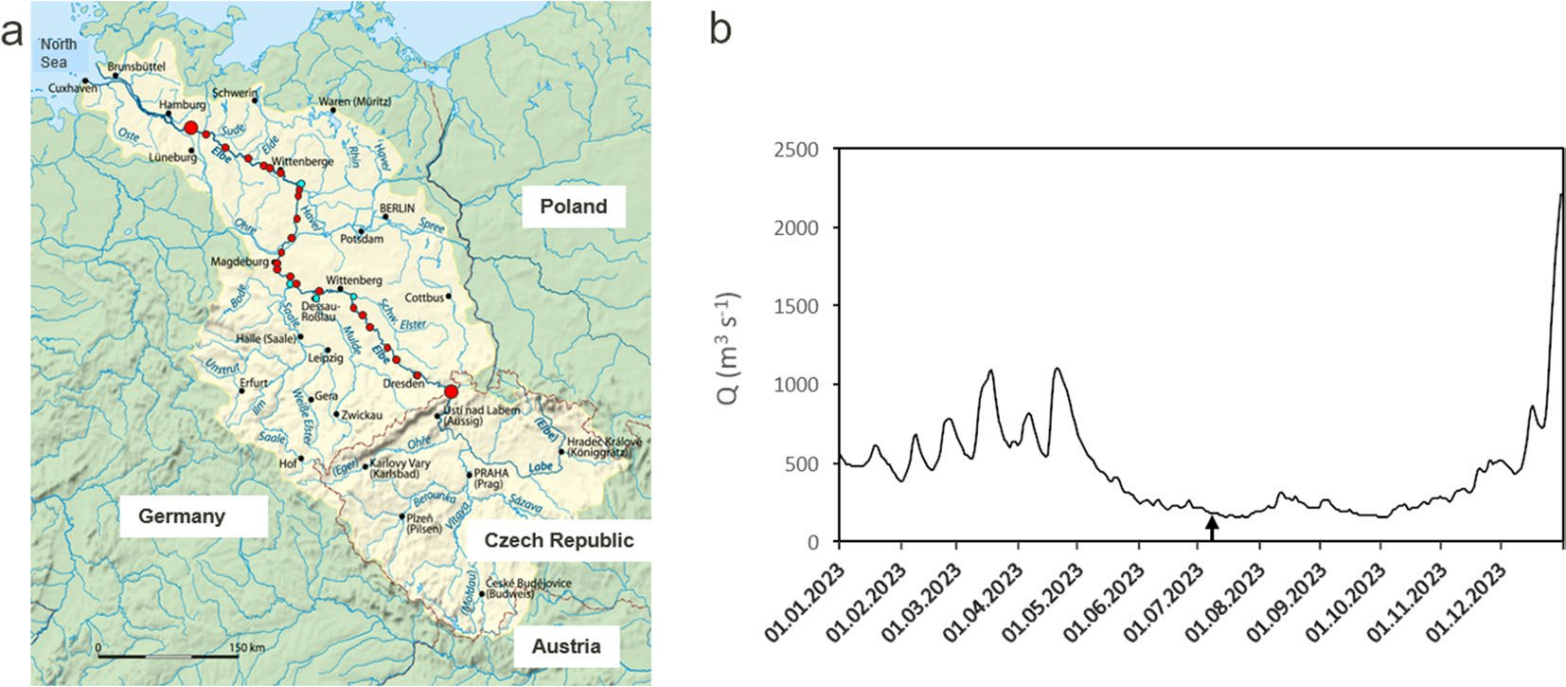
Map of the River Elbe catchment (https://de.wikipedia.org/wiki/Elbe) and sampling sites in the main stem (red) and tributaries (blue; **a**); discharge at gauge Magdeburg during 2023 with the arrow indicating Lagrangian sampling campaign (**b**)

Measurements were performed on the River Elbe in Central Europe which is 1094 km long from the Elbe source or 1252 km from the Vlatava source, respectively, and belongs to the 188 rivers of the world > 1000 km in length (Wikipedia, 2024). It has a catchment area of 148,268 km^2^ with 25 million inhabitants (ARGE-Elbe, 2001). The upstream part of the Czech Republic possesses several impoundments. We sampled the free-flowing middle part of the Elbe between Schmilka near the Czech–German border (4 km according to German river kilometrage) and the Geesthacht weir (585 km) which is near Hamburg before the tidal zone (Kamjunke et al., 2022). Four major tributaries flow into the Elbe along this stretch of river: Schwarze Elster, Mulde, Saale, and Havel (Fig. 1a). Using the research vessel Albis, we applied a Lagrangian approach, i.e., sampling of nearly the same water body along the way downstream according to its travel time (Kamjunke et al., 2022). The cruise was performed between 03 and 11 July 2023 at a low discharge of 186 m^3^ s^−1^ (Fig. 1b) which is less than the mean low discharge at Magdeburg in summer (231 m^3^ s^−1^). Using a horizontal sampler, samples were taken from 30 cm of water depth at the left bank, midstream, and right bank of each site.

Water chemical variables were measured using standard methods as described in Kamjunke et al. (2021): Samples were filtered onto quartz fiber filters (MN QR10, Macherey–Nagel) or glass fiber filters (GF-F, Whatman) immediately after sampling. Filtrates were stored at 4 °C until further analyses, and filters were frozen. We used the segmented flow technique for the photometric determination of nitrite (NO_2_^−^), nitrate (NO_3_^−^), and silicate (Si). The ammonium molybdate spectrometric method was used to measure total (TP) and soluble reactive phosphorus (SRP) after persulfate digestion. Chlorophyll *a* concentration was measured by high-performance liquid chromatography after calibration with commercial standards (Kamjunke et al., 2021). Dual nitrate isotopes were measured by the denitrifier method (Casciotti et al., 2002) (Sanders et al., 2018) where nitrate is converted to N_2_O which is measured with a GasBench II coupled to an isotope ratio mass spectrometer (Delta Plus XP, Thermo Fisher Scientific). The standard deviation for standards and samples was < 0.2‰ (*n* = 4) for the ẟ15N-nitrate and < 0.5‰ (*n* = 4) for the ẟ18O-nitrate.

The longitudinal sampling in 2023 was compared to eight other Lagrangian studies conducted during 2018–2022 (Kamjunke et al., 2021) (Kamjunke et al., 2022) (Kamjunke et al., 2023) (Hromova et al., 2024). Furthermore, we performed monthly routine monitoring from a bridge in Magdeburg (326 km) in 2018–2023. Samples were analyzed as described above. The results were used to compare data from 2023 with previous years and to detect temporal trends using linear regressions.

## Results and discussion

**Fig. 2:**
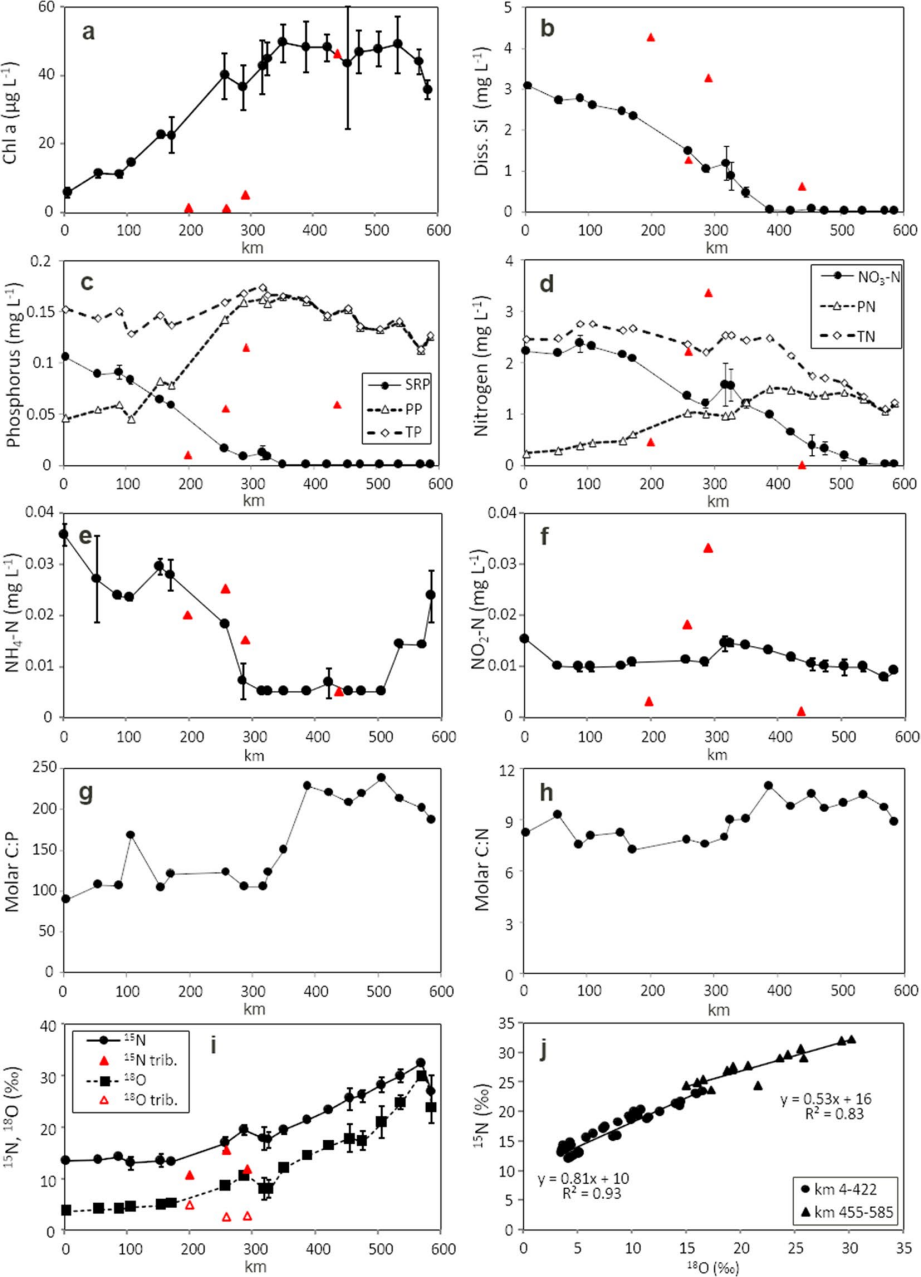
Longitudinal profiles of 2023 sampling of chlorophyll *a* (**a**), dissolved silicate (**b**), soluble reactive phosphorus (SRP), particulate phosphorus (PP) and total phosphorus (TP) concentrations (**c**), nitrate–N (NO_3_-N), particulate N (PN) and total N (TN) (**d**), ammonium-N (NH_4_-N) (**e**), nitrite-N (NO_2_-N) (**f**), molar C to P ratios of seston (**g**), molar C to N ratio of seston (**h**), and stable isotopes of nitrate (**i**) along the River Elbe (error bars, SD of three samplings; main tributaries indicated by red triangles). Relationship between nitrate ^18^O and nitrate.^15^N for the two river stretches (**j**)

The concentration of chlorophyll*a* increased from the beginning of the investigated stretch up to 350 km by a factor of 10, subsequently remained constant at a level of 50 µg L^−1^ until 535 km, and then decreased at the two most downstream sites (Fig. 2a) due to sedimentation (Kamjunke et al., 2022). It was low in the tributaries Schwarze Elster, Mulde, and Saale but high in River Havel. The growth of phytoplankton, i.e., the incorporation of nutrients for cell multiplication (Kamjunke et al., 2021), led to a decrease in concentrations of dissolved nutrients. The concentration of soluble reactive phosphorus decreased linearly until the detection limit at 350 km (Fig. 2c). Simultaneously, particulate phosphorus increased at a similar rate as the decrease in SRP so that the concentration of total phosphorus remained roughly constant. Phytoplankton increase stopped exactly at the site where SRP concentration reached the minimum detection limit, indicating that low phosphorus availability was the cause. This conclusion is supported by the molar carbon/phosphorus ratios of seston which were lower in the upstream part but increased to values around 200 in the downstream part (Fig. 2g). Those values are above the optimum Redfield ratio and regarded to indicate the beginning phosphorus limitation in phytoplankton (Hillebrand & Sommer, 1999).

Similar to SRP, the concentration of dissolved silicate also decreased (Fig. 2b), reaching the detection limit slightly later at km 388. Dissolved nitrogen was dominated by nitrate, whereas concentrations of nitrite and ammonium were two orders of magnitude lower (Fig. 2e and f). The concentration of nitrate also decreased along the river stretch (Fig. 2d). After a small maximum at 320 km, due to the inflow of the River Saale, it reached the detection limit at 570 km. Remarkably, the concentration continued to decrease after 350 km even though phytoplankton growth had already stopped. The concentration of particulate nitrogen increased until 388 km and remained constant further downstream. This rate of increase was less steep and lower than the rate of steep decrease of nitrate, leading to an overall decrease of total nitrogen, particularly from 388 km. The downstream decrease of nitrate and the loss of nitrogen might be solely explained by denitrification in the river as denitrification is the only process removing nitrogen in the form of nitrate permanently from rivers (Seitzinger et al., 2002). The molecular carbon/nitrogen ratio of seston was slightly higher in the downstream than in the upstream region (Fig. 1h) but below ratios for nitrogen limitation of phytoplankton.

**Fig. 3:**
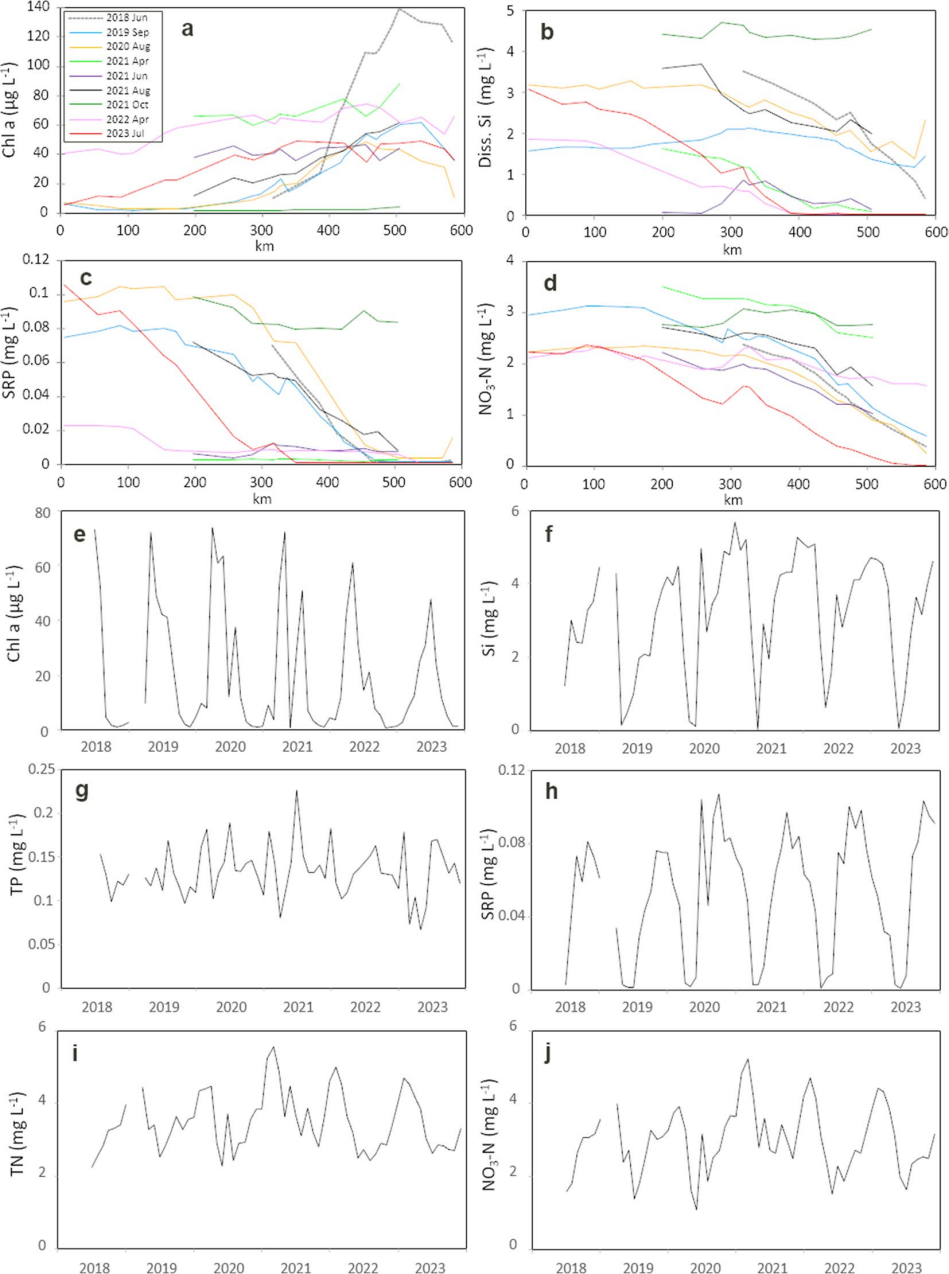
Comparison of 2023 to previous years: **a**–**d** longitudinal dynamics of chlorophyll *a*, dissolved silicate, soluble reactive phosphorus, and nitrate–N during nine studies between 2018 and 2023. **e**–**j** Temporal dynamics of chlorophyll *a*, dissolved silicate, total phosphorus (TP), soluble reactive phosphorus (SRP), total N (TN), and nitrate–N at Magdeburg (326 km) between 2018 and 2023 (monthly samplings)

The results of 2023 were compared to eight other Lagrangian samplings along the River Elbe. The concentration of chlorophyll *a* was intermediate in 2023, whereas higher values were observed in other years (Fig. 3a). SRP was reduced to low concentrations in several years (Fig. 3c), and dissolved silicate also showed very low downstream concentrations in some cases (Fig. 3b). In contrast, nitrate concentration decreased to the detection limit in 2023 for the first time, whereas concentrations were higher during all other samplings (Fig. 3d). However, the year 2023 did not have a particularly high chlorophyll *a* concentration. Thus, autotrophic nitrogen uptake may not explain nitrate exhaustion.

For a more representative analysis of the period 2018–2023, values of monthly samplings in Magdeburg were considered. Chlorophyll *a* concentration was highly dynamic, with maxima in spring and/or late summer and minima in winter (Fig. 3e). There was no temporal trend (*R*^2^ = 0.039), although the maxima of 2022 and 2023 were slightly lower. Concentrations of dissolved nutrients were also highly variable: dissolved silicate and SRP were reduced to very low concentrations in spring, whereas nitrate concentration was always > 1 mg L^−1^ in Magdeburg (Fig. 3f, h, and j). The overall nutrients (i.e., concentrations of total phosphorus and total nitrogen) remained constant over time, and none of the nutrients showed a significant temporal trend (*R*^2^, 0.0002–0.036). Thus, low nitrate concentrations in the downstream River Elbe may not be explained by a lower nitrogen level in the river.

Both stable isotopes of nitrate (^18^O and ^15^N) increased along the river stretch except the most downstream site (Fig. 2i). Maximum values of ^18^O and ^15^N were equal to those measured by Schulz et al. (2023). There was a positive relationship between the two isotopes with a higher slope in the upstream river section and a lower slope in the downstream part (Fig. 2j). During the nitrate reduction, the dual stable isotope signal of nitrate was enriched. The nitrate assimilation by phytoplankton typically results in a slope around 1 between ^18^O and ^15^N of nitrate (Granger et al., 2004), and the slope in the upstream part of the River Elbe was 0.81, indicating a dominance of autotrophic nitrate reduction. In contrast, nitrate reduction by denitrification results in a decoupled enrichment of ^18^O and ^15^N so that the slope differs significantly from 1 (Böttcher et al., 1990) (Granger & Wankel, 2016), and the slope in the downstream part of 0.53 is close to values of 0.5 typical for heterotrophic denitrification (Schulz et al., 2023). Thus, the ^18^O versus ^15^N enrichment in nitrate changed significantly which indicates a stronger impact of the denitrification than assimilation.

Denitrification may contribute considerably to N removal in rivers (Garnier et al., 2002), (Bouwman et al., 2005). A denitrification rate of 432 mg N m^−2^ d^−1^, as measured previously in the Elbe by Ritz et al. (2018), corresponds to a nitrate loss of 0.43 mg N L^−1^ over 3 days (assuming 3 m water depth) and might explain 45% of the nitrate decrease observed in our study between 388 and 585 km in 2023. This proportion in the very downstream part is higher than average values of 16% for stream denitrification across biomes (Mulholland et al., 2008). Schulz et al. (2023) reported a close coupling between river discharge and the riverine nitrogen cycle, with decreasing discharge and nitrogen loads declining disproportionally due to intensified nitrogen retention. Assimilatory nitrate uptake increased by enhanced phytoplankton growth due to long residence times and high light availability at low water levels (Schulz et al., 2023). But also, heterotrophic denitrification seemed to be promoted by low discharge: In the Elbe, this is particularly true in the downstream part where flow velocity decreases and sedimentation of organic material increases, leading to a decline of oxygen in sediments and enhanced production of methane (Bussmann et al., 2022). Low discharge and high temperatures promote the microbial process, and the rate of denitrification is higher under low discharge conditions than under high discharge conditions (Billen et al., 2007). As e result, the long-lasting drought in the Elbe catchment seemed to intensify riverine nitrate loss.

## Conclusion

We detected a longitudinal decline of phosphate, dissolved silicate, and nitrate below the detection limits along a large river which was observed for the first time in the case of nitrate. While the consumption of phosphate and silicate was due to phytoplankton uptake, the decrease of nitrate was a combined result of assimilatory nitrate uptake in the upstream part and heterotrophic denitrification in the downstream section. Further investigations should combine process measurements of autotrophic nutrient uptake, denitrification, and nutrient export in large rivers.

## Data Availability

The data of the longitudinal sampling in 2023 (except stable isotopes of nitrate) are available in Kamjunke, N., von Tümpling, W., Hoff, A., Koschorreck, M. (2023): Water chemistry of Lagrangian samplings of Inland Elbe 2023 (MOSES Hydrological Extremes). Helmholtz Centre for Environmental Research—UFZ, PANGAEA, 10.1594/PANGAEA.963359.
